# A Cytochrome P450 Conserved in Insects Is Involved in Cuticle Formation

**DOI:** 10.1371/journal.pone.0036544

**Published:** 2012-05-04

**Authors:** Tamar Sztal, Henry Chung, Silke Berger, Peter D. Currie, Philip Batterham, Phillip J. Daborn

**Affiliations:** 1 Department of Genetics, Bio21 Molecular Science and Biotechnology Institute, The University of Melbourne, Melbourne, Victoria, Australia; 2 Department of Biological Sciences, Monash University, Melbourne, Victoria, Australia; 3 Australian Regenerative Medicine Institute, Monash University, Melbourne, Victoria, Australia; University of Otago, New Zealand

## Abstract

The sequencing of numerous insect genomes has revealed dynamic changes in the number and identity of cytochrome P450 genes in different insects. In the evolutionary sense, the rapid birth and death of many P450 genes is observed, with only a small number of P450 genes showing orthology between insects with sequenced genomes. It is likely that these conserved P450s function in conserved pathways. In this study, we demonstrate the P450 gene, *Cyp301a1*, present in all insect genomes sequenced to date, affects the formation of the adult cuticle in *Drosophila melanogaster*. A *Cyp301a1 piggyBac* insertion mutant and RNAi of *Cyp301a1* both show a similar cuticle malformation phenotype, which can be reduced by 20-hydroxyecdysone, suggesting that *Cyp301a1* is an important gene involved in the formation of the adult cuticle and may be involved in ecdysone regulation in this tissue.

## Introduction

Cytochrome P450s are an evolutionarily ancient gene family found in virtually all organisms [1]–[5]. P450s were originally characterised for their roles in the detoxification of xenobiotics, but further studies have shown that some P450s possess catalytic roles in the metabolism of many essential endogenous molecules [6]–[8]. In vertebrates, it has been suggested that evolutionarily conserved P450s function in endogenous pathways while those that are poorly conserved between species arise as evolutionary responses to xenobiotic challenges [9].

In insects, the number of cytochrome P450s in sequenced genomes ranges from 37 in the body louse, *Pediculus humanus* [10], to 160 in the dengue mosquito, *Aedes aegypti* [11]. Throughout evolution, selection results in tailoring an organism's genome. Thus genes involved in essential developmental processes are likely to be retained whereas those involved in specific detoxification responses, depending on an organism's environment, may not be under the same functional constraints. Some P450s are highly conserved and considered stable, with single orthologs found between species for those genes with proven biosynthetic and/or housekeeping roles [9]. Of the P450s conserved between *D. melanogaster* and *A. aegypti* [11], *Cyp302a1*, *Cyp306a1*, *Cyp307a1*, *Cyp307a2*, *Cyp314a1*, *Cyp315a1* and *Cyp18a1*, are involved in the biosynthesis, activation and inactivation of the essential growth hormone 20-hydroxyecdysone [12]–[19], a conserved pathway found in all insects. *Cyp4g1*, which is involved in lipid metabolism [20] and *Cyp4g15*, which is expressed in the brain and central nervous system [21] are also both highly conserved in distant insect genomes.

In this study, we investigate *Cyp301a1*, another P450 found in all sequenced insect genomes to date. A previous study has shown that *Cyp301a1* is expressed in both the embryonic and larval hindgut as well as the embryonic epidermis of *D. melanogaster* [22]. Here we show that *Cyp301a1* is likely to possess an important function in the developing epidermis. RNAi knockdown of *Cyp301a1,* and a *Cyp301a1 piggyBac* element insertion mutant both result in adults with a cuticle defect. Histological analyses suggest a retention of larval epidermal cells down the central portion of the abdomen. As *Cyp301a1* is a conserved P450 in insects, further investigations into *Cyp301a1* function may reveal critical biochemical insights into the formation of the adult cuticle.

## Materials and Methods

### Phylogenetic analysis of Cyp301a1

The *D. melanogaster* Cyp301A1 predicted protein sequence was used as a query against representative databases from selected Diptera, Lepidoptera, Coleoptera, Hymenoptera and Phthiraptera species using a BLASTp search (NCBI; http://flybase.bio.indiana.edu/blast). Corresponding orthologs to both Cyp301A1 and Cyp301B1 were identified and annotated using Artemis [23]. The sequences were aligned using ClustalX [24] and a maximum likelihood tree (bootstrapping  = 1000) was compiled using MEGA 5.05 [25]. *D. melanogaster* Cyp49A1 was used as an outgroup.

### *Drosophila* stocks and vectors

The D. melanogaster stocks y; cn, bw; sp (stock number 2057), y^1^ w^*^; P{tubP-GAL4}LL7/TM3, Sb^1^ (stock number 5138), the piggyBac transposase stock w^1118^; CyO, P{Tub-PBac\T}2/wg^Sp-1^ (stock number 8285) and the Cyp301a1 piggyBac insertion stock w^1118^; PBac{w^+mC^ = WH}Cyp301a1^f02301^ (stock number 18537) were obtained from the Bloomington Drosophila Stock Center, Indiana. All stocks were maintained on glucose, semolina and yeast medium at 25°C. Drosophila transformation vectors were obtained from the Drosophila Genomics Resource Center (DGRC), Indiana.

### Synthesis of cDNA, real-time PCR and *in situ* hybridisation

Total RNA was extracted using TRIzol reagent (Invitrogen Life Technologies). RNA samples were treated with RQ1 RNase-free DNase (Promega). cDNA was synthesised from 2 µg of each RNA sample in a 20 µl reaction using Superscript III Reverse Transcriptase (Invitrogen Life Technologies) and oligo(dT)_20_ primer following the supplier's instructions. Quantitative PCR (QPCR) was performed as previously described using *RpL11* as a housekeeping gene [26]. Primers for QPCR of *Cyp301a1* were *Cyp301a1*-RT-F (5′-ACCGCGAATACACTCCACTT-3′) and *Cyp301a1*-RT-R (5′-TGGCATCAGTCTCCATGTATT-3′). For *in situ* hybridisation, PCR was performed on cDNA using primers spanning the complete *Cyp301a1* open reading frame (ORF) (*Cyp301a1-*ORF-F ATGAACAATCTGTCGCTGAAAGCTTGG and *Cyp301a1-*ORF-R CTAAACTCGTGTCATCTTAAAGCGCAG) and the PCR product was cloned into pGEM-T Easy (Promega). DIG-labeled RNA probe synthesis and *in situ* hybridisation were performed as previously described [22], [27]. *In situ* hybridisations were performed on the *y; cn bw sp* strain. Primers for *Cyp4g1* and *Cyp6g1* were previously reported [22], [26]. For adult integument RNA preparations, the integument was carefully removed from the inner abdominal layer and cDNA was synthesised using previously described methods.

### RNAi knockdown experiments

The UAS-*Cyp301a1*-RNAi lines were constructed using the pWIZ vector [28]. BLASTn searches were performed on the ORF of *Cyp301a1* to determine regions of similarity to other genes in the *D. melanogaster* genome. A region was chosen that contained less than 17 base pair sequence similarity to any other gene (to avoid off-targets) and that spanned between 300–500 base pairs. The sequence GGCCTCTAGA (which contains an *Xba*I cleavage site) was added to the end of both primers. Primers used were 5′-AAAGCTCCCTATTGGAGATACTTT-3′ and 5′-GTAGTCGCTTTAATTCCTCGTGAAC-3′. The fragment was amplified by PCR from cDNA using Expand High Fidelity^PLUS^ PCR system and cloned into *pGEM-T Easy* to confirm the correct sequence. The fragment was digested with *Xba*I before being cloned into *pWIZ* via the *Avr*II site. The construct was sequenced to determine the orientation of the inserted fragment before the second fragment was cloned into the *Nhe*I site. PCR and restriction digest confirmed that the second fragment to be cloned in the opposite orientation to form a hairpin. Transformation into *D. melanogaster w^1118^* strain was performed using standard techniques.

### Histological analyses

Adult female flies were taken within 24 hours of eclosion, partially dissected and fixed in formalin for at least eight hours at room temperature before being embedded in paraffin. Sections were performed using the cut-4060 microtome (Microtec) at 15 μm. Transverse sections were taken through the disrupted portion of the cuticle in *Cyp301a1* ^f02301^ flies and corresponding regions in controls. Sections were mounted on poly-lysine slides and either stained with Hematoxylin and Eosin or Calcofluor White (Sigma). Tissues were visualised using a Zeiss AxioImager Z1 microscope and a Leica SP5 confocal.

### Excision of piggyback element from *Cyp301a1* ^f02301^

*Cyp301a1* ^f02301^ females were crossed to males from the piggyBac transposase strain *w^1118^; CyO, P{Tub-PBac\T}2/wg^[Sp-1]^*. F_1_ males (genotype *w^1118^*; *Cyp301a1^f02301^/CyO, P{Tub-PBac\T}2* were then crossed to a double balancer strain (*w^1118^; If/CyO; TM3, Sb/TM6B, Tb*) to isolate transposition events and identified by progeny with white eyes. These flies were then made homozygous for the second chromosome (where *Cyp301a1* is located) and analysed by PCR.

### 20-hydroxyecdysone exposure assays

Third instar larvae of the *Cyp301a1* ^f02301^ strain were exposed to 1 mg/ml 20-hydroxyecdysone (20E) (Enzo Life Sciences) in instant fly media formula 4–24 (Carolina Biological Supply Company) as described previously [29]. Ten replicates of 10 larvae per vial were exposed to 20E, and the abdomen phenotype of all emerging flies was scored. Controls were reared under the same conditions but in the absence of 20E.

## Results

### *Cyp301a1* is evolutionarily conserved within insect species

**Figure 1:**
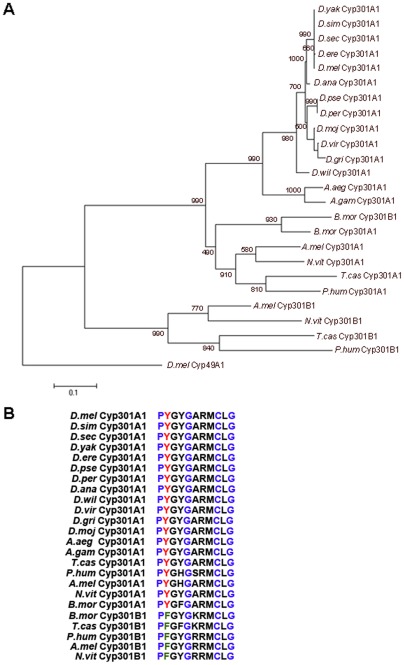
*Cyp301a1* is a conserved insect P450. (**A**) Phylogenetic analysis of Cyp301A1 and Cyp301B1 in the insect species. Values shown represent 1000 bootstrapping values. *D. melanogaster* (*D.mel*) Cyp49A1 was used as an outgroup. *D.ere*  =  *D. erecta, D.sec*  =  *D. sechellia*, *D.sim  =  D. simulans*, *D.yak*  =  *D. yakuba*, *D.ana*  =  *D. ananassae*, *D.per*  =  *D. persimilis*, *D.pse*  =  *D. pseudoobscura*, *D.gri*  =  *D. grimshawi*, *D.vir*  =  *D. virilis*, *D.moj* = *D. mojavensis*, *D.wil*  =  *D. willistoni*, *A.gam*  =  *A. gambiae*, *N.vit*  =  *Nasonia vitripennis*, *A.mel*  =  *Apis mellifera T.cas*  =  *Tribolium castaneum*, *B*.*mor*  =  *Bombyx mori*, *P.hum*  =  *Pediculus humanis*. (**B**) Alignment of the heme-binding domain of Cyp301A1 and Cyp301B1 from representative insect species. Conserved residues in the heme-binding domain consensus sequence (PxxxGxxxCxG) are highlighted in blue. The conserved Phenylalanine (F) in the second position (highlighted in green) in Cyp301B1 orthologs is changed to a Tyr (Y) (highlighted in red) in all Cyp301A1 orthologs.

To identify orthologs of *D. melanogaster Cyp301a1*, the predicted protein sequence was used to query the genomes of twelve *Drosophila* species as well as other related insect species. A single *Cyp301a1* ortholog was found in each genome of the 12 *Drosophila* species with available sequence [30], as well as in representative genomes from sequenced insect orders [31]–[33]. Given the rapid evolution of most members of the P450 family, this conservation indicates the gene product has an important function in all insects. The inferred Cyp301 protein sequences were aligned and a phylogenetic analysis was performed. The tree shows an ancient duplication of the *Cyp301* gene family prior to the divergence of the *Phthiraptera* species forming the *Cyp301a1* and *Cyp301b1* genes and then a subsequent loss of the *Cyp301b1* copy in Diptera (Figure 1A). Interestingly, Cyp301A1 in all insect species analysed has a Tyr (Y) instead of the highly conserved Phe (F) present in the second position of the heme-binding domain (PFxxGxxxCxG) (Figure 1B). A Tyr in this position is not observed for any other *D. melanogaster* P450 [1]. Cyp301B1 orthologs do not possess this change in the heme-binding domain, suggesting some functional divergence between the *Cyp301* genes following their duplication.

### Temporal and spatial expression of *Cyp301a1* during *D. melanogaster* development

**Figure 2:**
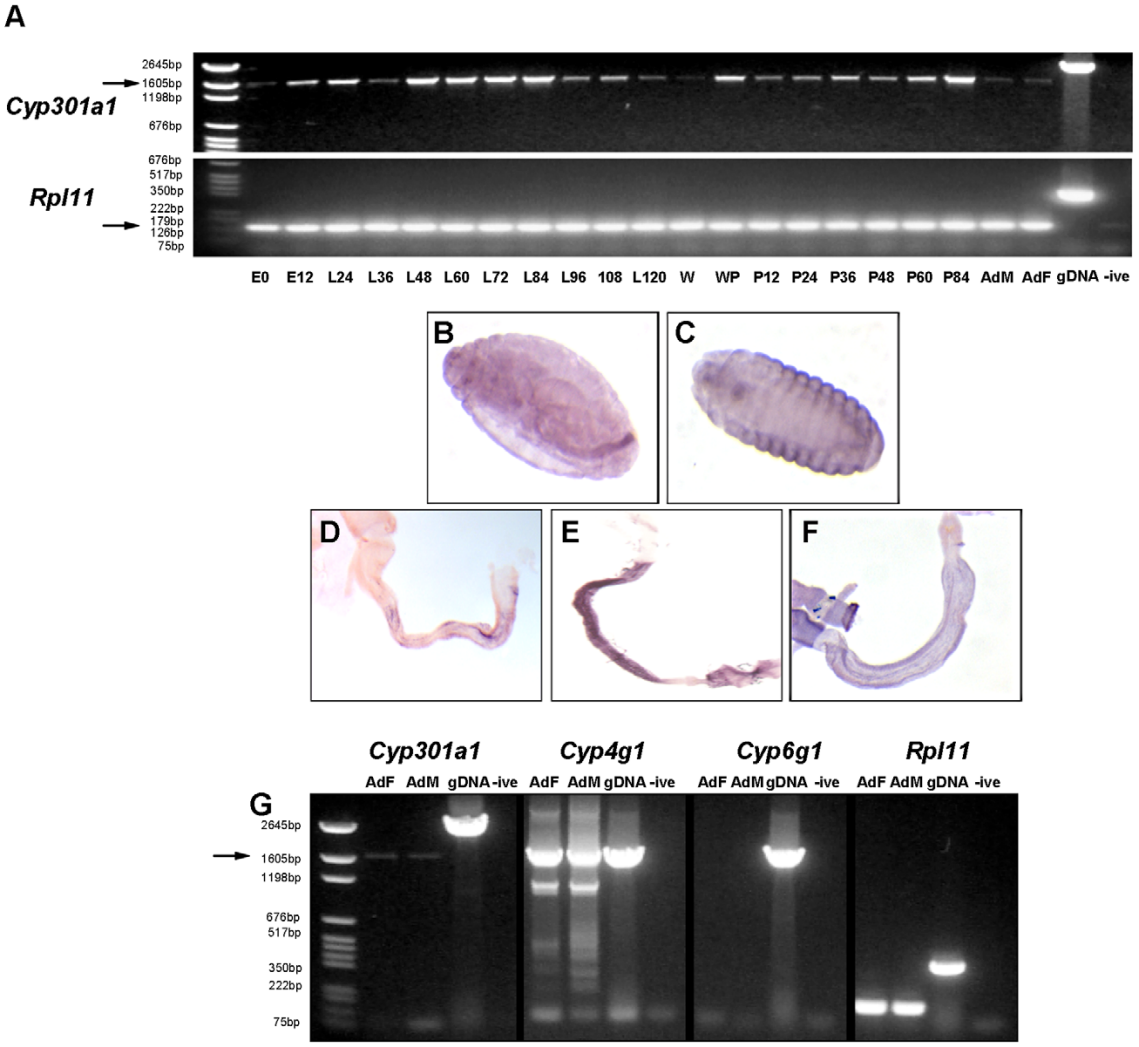
Expression of *Cyp301a1* in *D. melanogaster*. (**A**) RT-PCR analysis of *Cyp301a1* expression during the *D. melanogaster* development (expected size; cDNA = 1659 bp (arrow), gDNA = 2596 bp). Developmental stages are listed across the bottom of the gel in hours (E = embryo, L = larvae, W = wandering third instar, WP = white pupal stage, P =  pupae, AdF  =  adult female, AdM  =  adult male). Animals were re-staged at onset of pupation. *RPL11* was used as a housekeeping control gene and gDNA as a genomic DNA control (expected size; cDNA = 141 bp (arrow), gDNA = 355 bp). (**B–F**) *In situ* hybridisation of *Cyp301a1.* (**B**) At embryonic stages 13–16, *Cyp301a1* is detected in the hindgut and (**C**) epidermis. *Cyp301a1* is detected in the hindgut during (**D**) third instar larval, (**E**) pupal and (**F**) adult stages. (**G**) RT-PCR analysis of genes expressed in the adult integument. *Cyp301a1* is detected in the adult female (AdF) and male (AdM) integument (arrow). *Cyp4g1* (highly expressed in oenocytes which were included in integument dissections) is used as a positive control. *Cyp6g1* is not detected in the integument samples (Chintapalli et al., 2007) and used as a negative control.

*Cyp301a1* was detected by reverse transcription (RT)-PCR at all life stages, from embryo to adult (Figure 2A). *Cyp301a1* expression is high during second instar larval stage (from L48), but decreases during the late third instar larval stage (L96-L120) and is essentially absent in wandering (W) larval stages (Figure 2A). *Cyp301a1* expression then increases shortly during pupal formation (WP) and then again during late pupal development prior to eclosion (Figure 2A).

To examine the spatial expression of *Cyp301a1*, *in situ* hybridisation was performed on various stages during *D. melanogaster* development. *Cyp301a1* expression in stage 1–2 embryos, the embryonic hindgut (stage 12–17) and epidermis (stage 17), as well as the larval hindgut has previously been characterised [22]. These expression patterns have been confirmed, and additional *Cyp301a1* expression has been detected in the pupal and adult hindgut (Figure 2B–F). *Cyp301a1* expression has also been reported in the larval trachea and larval carcass [34]. In addition, we detected *Cyp301a1* expression by RT-PCR using cDNA synthesised from dissected adult integument (Figure 2G). To confirm the validity of this expression we also tested the expression of *Cyp4g1* (known to be expressed in the oenocytes residing in the epidermis [22]), *Cyp6g1* (a gene primarily expressed in the midgut, Malphigian tubules and fat body [26]) and the *Rpl11* housekeeping gene (Figure 2G). As expected, *Rpl11* and *Cyp4g1* were expressed highly in both male and female integument samples whereas *Cyp6g1* could not be detected from either cDNA sample (Figure 2G).

### A piggyBac insertion in *Cyp301a1* results in malformation of the adult cuticle

**Figure 3:**
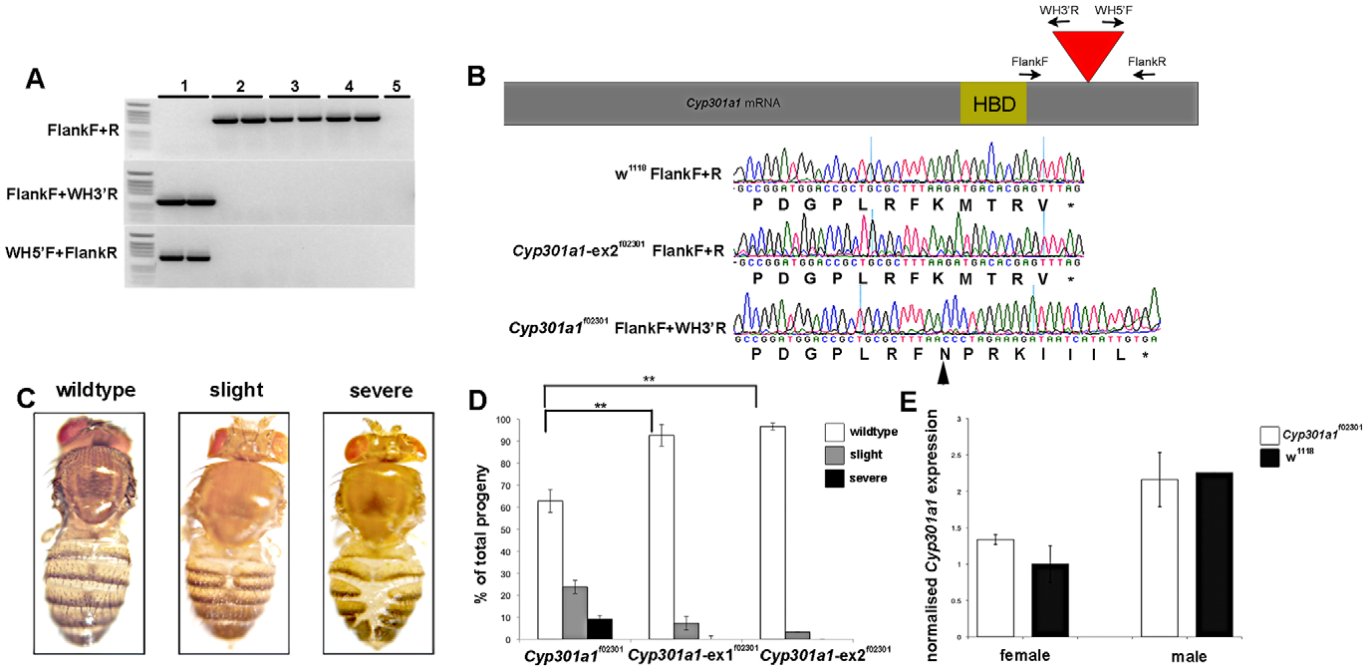
Characterisation of the *Cyp301a1* ^f02301^ *piggyBac* insertion strain. **(A)** PCR screen for the presence or absence of the *piggyBac* element in the *Cyp301a1^f02301^* and excised (*Cyp301a1*-ex *^f02301^* lines). There is no amplification of WH5′/3′ primers from lines without the *piggyBac* element. 1 =  *Cyp301a1^f02301^*, 2 = w118, 3 =  *Cyp301a1-ex1^f02301^*, 4 = *Cyp301a1ex-2^f02301^*, 5 = negative control) (**B**) Scheme of the *Cyp301a1* mRNA sequence indicating the position of the *piggyBac* element in the *Cyp301a1* gene (red triangle) just following the heme binding domain (HBD). The primers used for screening of the *piggyBac* insert are represented on the scheme. Below, chromatograms showing the base changes in the *Cyp301a1^f02301^* strain (bases following black arrowhead) due to the *piggyBac* insertion compared to the *w^1118^* background strain and excised lines. (**C**) The cuticle phenotype observed in the *Cyp301a1* ^f02301^ strain was divided into three categories based on phenotypic severity. (**D**) Quantification of the proportion of flies emerging from the *Cyp301a1^f02301^* and *Cyp301a1*-excised *^f02301^* lines according to the categories outlined in (C). **p<0.01 (**E**) Quantitative RT-PCR analysis of *Cyp301a1* mRNA levels in *Cyp301a1* ^f02301^ and *w^1118^* adult flies (collected at eclosion). Values are relative to *RPL11*.

A transgenic *D. melanogaster* line, *Cyp301a1* ^f02301^ (*PBac{WH}Cyp301a1^f02301^*), contains a *piggyBac* element inserted within the *Cyp301a1* predicted open reading frame [35]. Sequencing of the *Cyp301a1* ^f02301^ allele using primers specific to the *piggybac* element confirmed the presence of the WH-*piggyBac* element insertion, predicted to change the final five amino acids of the Cyp301A1 protein (Figure 3A, B). These changes are predicted to extend the C-terminal end of the protein by three amino acids and to increase the hydrophobicity of the protein, possibly altering the protein structure (Figure 3B). It is unknown if this results in a complete loss of function of the Cyp301A1 protein. Analysis of *Cyp301a1* ^f02301^ flies revealed a cuticle phenotype in both males and females, ranging from a slight malformation of the cuticle between the tergites to a complete loss of the tanned cuticle layer down the dorsal midline of the abdomen. The banding on the abdomen appeared severed, causing a misalignment of symmetry between the tergites. The range of phenotypes observed was classified into three categories; those that were wildtype (no visible disruption to the cuticle), slight (slight tearing of the cuticle down the mid-line) and severe (complete cuticle disruption) (Figure 3C). The *Cyp301a1* ^f02301^ flies also showed reduced survival with only 80% of larval forming pupae and of these, only 90% eclose to adults. Of the emerging progeny reared at 25°C, 63(±5)% showed no phenotype, 24(±4)% of progeny emerged with a slight cuticle phenotype and 10(±1.5)% of progeny emerged with a severe cuticle disruption (Figure 3D). We measured the *Cyp301a1* mRNA levels by QPCR in *Cyp301a1* ^f02301^ adult flies (collected at eclosion) and found no significant difference compared to the background *w^1118^* strain (Figure 3E), although we did detect higher *Cyp301a1* expression in males than females. This suggests that the insertion is likely to affect *Cyp301a1* protein stability or structure, rather than transcription.

To determine whether the cuticle phenotype seen in the *Cyp301a1* ^f02301^ line was due to a disruption of the *Cyp301a1* gene, the *piggyBac* element was excised (Figure 3A). Two independent excised lines (*Cyp301a1*-ex1a^f02301^ and *Cyp301a1*-ex2a^f02301^) were obtained, which were screened by PCR and sequenced to confirm the presence of a functionally wild-type *Cyp301a1* gene (Figure 3B). Unlike other eukaryotic class II transposons, *piggyBac* excisions are precise and do not leave a footprint [36]. Phenotypic analysis of the *Cyp301a1*-ex^f02301^ flies raised at 25°C showed a significant decrease in appearance of the cuticle malformation phenotype and increase in the wild type phenotype compared to the *Cyp301a1* ^f02301^ line (Figure 3D).

### RNAi of *Cyp301a1* results in similar cuticle phenotypes as *Cyp301a1* ^f02301^ lines

**Figure 4:**
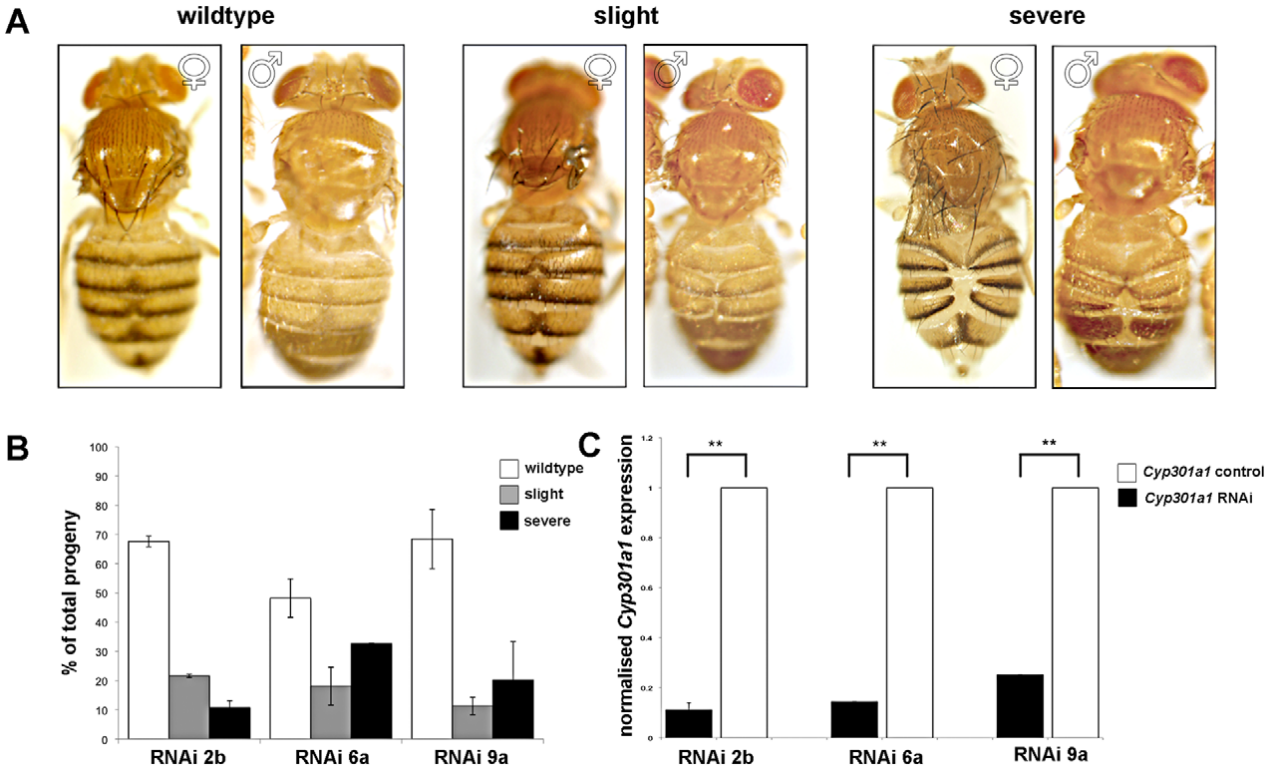
Characterisation of the phenotype observed in *Cyp301a1* RNAi progeny. **(A)** Phenotypes of *Cyp301a1* RNAi driven by the *tubulin-*GAL4 driver were characterised as described in Figure 3. Both males and females are shown. (B) Quantification of the proportion of flies emerging from the *Cyp301a1* RNAi lines, according to the categories outlined in A. (C) Quantitative RT-PCR of *Cyp301a1* in three independent *Cyp301a1* RNAi lines (2b, 6a and 9a). Values are relative to *RPL11* and normalised to *Cyp301a1* expression in each of the controls (*Cyp301a1* control). **p<0.01.

To further confirm that *Cyp301a1* was causing the malformed cuticle phenotype, we constructed three independent RNAi lines targeted against *Cyp301a1*. When *Cyp301a1* was silenced using the ubiquitous *tubulin*-GAL4 driver y^1^ w^*^; P{tubP-GAL4}LL7/TM3, Sb^1^ [37] and progeny were raised at 25°C, all *Cyp301a1* RNAi progeny showed a similar range of cuticle phenotypes as seen in the *Cyp301a1* ^f02301^ flies (Figure 3A). QPCR on RNA isolated from adult males at eclosion showed that *Cyp301a1* expression was significantly decreased (70–90%) in the *Cyp301a1* RNAi progeny compared to controls (Figure 4C). Akin to the previous phenotypic classifications used, *Cyp301a1* adult progeny were classed in order of cuticle phenotype severity (Figure 4A). At 25°C, 61(±7)% of flies emerged with no phenotype, 17(±3)% of RNAi progeny emerged with a slight cuticle malformation and 22(±7)% of RNAi progeny emerged with a severe cuticle phenotype from all three lines (Figure 4B). The cuticle phenotype was equally prevalent in males and females (Figure 4A). These RNAi results corroborate those reported for the *Cyp301a1* ^f02301^ insertion strain, suggesting that a loss of *Cyp301a1* is likely to be responsible for the cuticle malformation.

### *Cyp301a1* ^f02301^ flies show a cellular defect in adult cuticle formation

**Figure 5:**
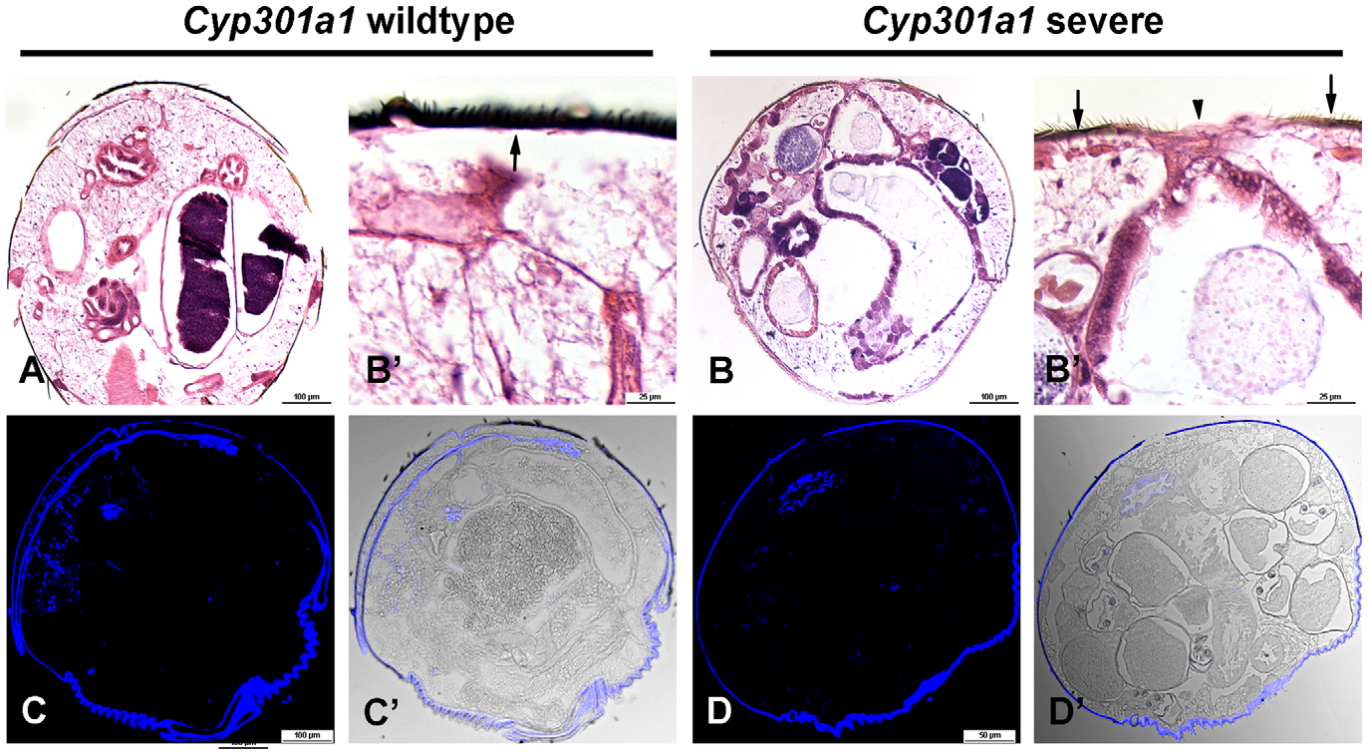
Histological analysis of *Cyp301a1^f02301^* flies showing a severe cuticle phenotype. (**A-B**) Hematoxylin and eosin staining of sections. (A and enlarged in A′) Transverse section through the abdomen of a *Cyp301a1* control sample shows a normal, pigmented cuticle layer lining the abdomen (arrow). (B and enlarged in B′) Transverse section through the abdomen of a *Cyp301a1^f02301^* sample shows a central unpigmented layer, in the region where the cuticle is disrupted (arrowhead). The surrounding cuticle looks wild type (arrows). (C) Calcofluor staining (blue; overlayed with brightfield in C′) of transverse sections from *Cyp301a1* control samples showing staining across the cuticle. (D) Calcofluor staining (blue; overlayed with brightfield in D′) of transverse sections from *Cyp301a1^f02301^* samples showing staining across the cuticle. but is absent from the central regions where the cuticle is disrupted.

To further understand the cellular pathology observed in the *Cyp301a1* ^f02301^ flies, transverse sections of the abdominal cuticle at eclosion were stained with hematoxylin and eosin. Although there is a lack of cells in the central region, there appears to be an intact cuticle layer (albeit thinner and nonpigmented), which spans the two sides of the tergites in *Cyp301a1* ^f02301^ flies (Figure 5B,B′). Calcofluor staining shows that chitin is not secreted in these areas, although the remaining cuticle appears correctly pigmented, similar to control sections (Figure 5C,D). Compared to control flies, where transverse sections clearly show an even distribution of abdominal sensory bristles lining the cuticle layer (Figure 5A,A′), in *Cyp301a1* ^f02301^ flies there appears to be a lack of abdominal sensory bristles in regions surrounding the central cavity, where this cuticle is improperly formed (Figure 5B,B′). The lack of sensory bristles in this central region may suggest a failure of proper histoblast migration, given that abdominal histoblasts are also responsible for correct formation of sensory bristles along the abdomen.

### The *Cyp301a1* cuticle malformation phenotype can be reduced by 20-hydroxyecdysone

20E pulses during insect development define periods of growth and metamorphosis [38]. During larval stages, imaginal discs express high levels of Ecdysone Receptor (EcR), which when bound by 20E, is involved in the transcriptional activation of many genes [39]. 20E is likely to mediate both the destruction of unwanted larval tissues and simultaneous differentiation of adult tissues [40]. It was shown recently that an early peak of 20E during the prepupal stage activates the synchronous division of histoblasts while a later peak is essential for larval cell replacement [41].

**Figure 6:**
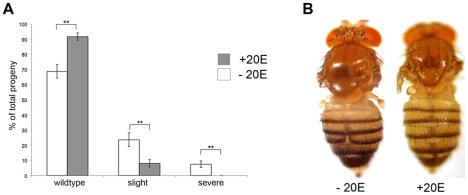
Rescue of *Cyp301a1^f02301^* flies using 20E. (A) Quantification of the proportion of flies emerging from the *Cyp301a1^f02301^* line with or without 20E treatment, according to the categories outlined in Figure 3A. **p<0.01. (B) Phenotypes of *Cyp301a1^f02301^* flies at eclosion with or without 20E treatment.

To determine whether the cuticle phenotype observed in the *Cyp301a1* RNAi and *Cyp301a1* ^f02301^ flies was dependent on 20E, flies were raised on a diet supplemented with 20E. *Cyp301a1* ^f02301^ larvae fed 20E during development eclosed with a significantly reduced incidence of the severe and mild abdominal phenotype, and significantly increased incidence of the wild type cuticle phenotype when compared with those fed control food (Figure 6). This suggests that *Cyp301a1* may be involved in ecdysone regulation during adult cuticle formation.

## Discussion

Insect cytochrome P450s are a large gene family, with roles in development and xenobiotic detoxification [1], [42]. Despite the large numbers of P450s found in insects, the number of orthologous P450s conserved between insects is small. In general, it has been observed that conserved P450s have developmental roles, such as the P450s involved in 20E biosynthesis in *D. melanogaster* and *Manduca sexta*, [43]–[45]. The conservation of *Cyp301a1* in insects indicates that *Cyp301a1* is likely to play an important role. Phylogenetic analyses show that *Cyp301a1* possesses a restricted pattern of evolution, typical for many stable P450s [9]. A single *Cyp301a1* ortholog is present in 12 *Drosophila* species with sequenced genomes [30]. Interestingly the Tyr to Phe change, conserved in the heme-binding domain of CYP301A1 orthologs is not found in CYP301B1 sequences, the nearest ortholog. Although sequence changes in the conserved heme-binding domain of P450s are rare, they are characteristic for P450s that act as atypical monooxygenases, for example CYP74A, a plant allene oxide synthase, and CYP5A1, the vertebrate thromboxane synthase enzyme [1], [46].

*Cyp301a1* is expressed in a number of tissues throughout *D. melanogaster* development. It is expressed in the larval, pupal and adult hindgut, the epidermis of stage 17 embryos and in the larval trachea and carcass [34]. Disruption of *Cyp301a1* function, as demonstrated by both *Cyp301a1 piggybac* and RNAi knockdown experiments, produced adult flies with a distinct morphological disruption to the cuticle. Histological analyses revealed an improper fusion of tergites along the abdomen, which may be caused by an abnormal proliferation of the adult cuticle. This phenotype is significantly reduced in the *Cyp301a1* RNAi control groups, and the *Cyp301a1*-ex^f02301^ strains, suggesting that the cuticle malformation is specifically due to a disruption in *Cyp301a1* function.

The cuticle malformation phenotype, although replicable in both genotypes, showed incomplete penetrance with approximately 60% of flies affected in each case. This incomplete penetrance could be due to variability in the loss of *Cyp301a1.* RNAi of *Cyp301a1* results in a decrease of *Cyp301a1* mRNA levels of 70–90% as measured by QPCR, and it is not known if the *Cyp301a1* piggyBac insertion completely abolishes CYP301A1 function. A more extreme phenotype may result if a *Cyp301a1* null allele is created. Alternatively, the incomplete penetrance could result from a redundancy in *Cyp301a1* function. Several other P450s, such as *Cyp49a1* and *Cyp18a1* are expressed in the integument during Drosophila development [19], [22]. Although *Cyp301a1* is highly conserved and thus likely to possess an essential function, additional P450s may be able to partially compensate for the reduced function of *Cyp301a1* in the *Cyp301a1* ^f02301^ and *Cyp301a1* RNAi flies.

A similar abdominal phenotype has been documented and is caused by a lack of larval epidermal cell (LEC) replacement, which obstructs the closure of the adult abdominal epithelium [29], [41]. Usually, newly formed adult abdominal tergites arise during metamorphosis as polyploid LECs are replaced by the descendants of the histoblasts imaginal cells, derived from small lateral nests in the larva. The histoblasts divide and migrate dorsally and ventrally over the abdomen until its whole surface is covered with cells [41], [47]–[48]. During this process, the LECs undergo apoptosis; they constrict apically, are extruded from the epithelium and are subsequently phagocytosed [41]. Sustained expression of *Activating transcription factor 3* (*Atf3*) alters the adhesive properties of LECs, thus preventing their extrusion and replacement by the adult epidermis [29]. Removal of LECs is normally complete by 36 hours after pupal formation, at which time sheets of histoblasts reach the dorsal midline [49]. *Atf3* expression is high during early larval development and is downregulated during early pupal formation with a late peak of expression just prior to eclosion [49], a similar expression profile to *Cyp301a1.* Retention of LECs caused by sustained *atf3* expression appears to be Jun-mediated and can be partially rescued with the addition of 20E [29]. Similarly, we also supplied ecdysone via this feeding method [29] to *Cyp301a1* ^f02301^ flies, which seems to allow progression through development without inhibiting adult ecdysis (as seen when 20E is administered via injection). Survival could be seen because the actual concentration that is entering larvae (assuming the larvae are only exposed when feeding, and not at later life stages) was not high enough to alter metamorphosis, and this concentration is not maintained during pupal development. Our experiments show that 20E exposure during third instar larval stage partially rescues the abdominal closure defects in *Cyp301a1* ^f02301^ flies, possibly due to altering the early 20E peak. 20E is also involved in activating several genes in the chitin biosynthesis pathway, forming the polysaccharide layer in the cuticle [50]. However, given that intact regions of chitin are present in the *Cyp301a1* RNAi and *Cyp301a1* ^f02301^ flies, it is unlikely that CYP301A1 is involved in chitin synthesis.

Although we cannot conclude that CYP301A1 is directly involved in 20E regulation, it is not unprecedented for P450s to be involved in 20E regulation during development. *Cyp302a1*, *Cyp306a1*, *Cyp307a1*, *Cyp307a2*, *Cyp314a1* and *Cyp315a1* are all involved in the biosynthesis and activation of the essential growth hormone 20-hydroxyecdysone [12]–[18] while *Cyp18a1* is involved in the inactivation of 20E [19]. Mutants in *Cyp18a1* affect the timing and shaping the 20E peaks during metamorphosis [19]. As the inactivation of ecdysteroids possibly involves more than one enzyme, with ecdysteroid metabolic breakdown products located in the gut and other tissues [51], it is possible that *Cyp301a1* is somehow involved in ecdysteroid metabolism, or perhaps some other aspect of 20E signalling or regulation during adult cuticle formation.
